# One-Component Catalytic Electrodes from Metal–Organic Frameworks Covalently Linked to an Anion Exchange Ionomer

**DOI:** 10.3390/molecules30061230

**Published:** 2025-03-10

**Authors:** Riccardo Narducci, Emanuela Sgreccia, Alessio Vincenzo Montella, Gianfranco Ercolani, Saulius Kaciulis, Suanto Syahputra, Emily Bloch, Luca Pasquini, Philippe Knauth, Maria Luisa Di Vona

**Affiliations:** 1 Tor Vergata University of Rome, Department Industrial Engineering and International Laboratory-Ionomer Materials for Energy, 00133 Roma, Italy; emanuela.sgreccia@uniroma2.it (E.S.); alessiovincenzo.montella@alumni.uniroma2.eu (A.V.M.); 2 Chemistry Department, Tor Vergata University of Rome, Via della Ricerca Scientifica, 00133 Roma, Italy; ercolani@uniroma2.it; 3 Institute for the Study of Nanostructured Materials, ISMN-CNR, Monterotondo Stazione, 00015 Roma, Italy; saulius.kaciulis@ismn.cnr.it; 4 Aix Marseille University, CNRS, MADIREL (UMR 7246) and International Laboratory-Ionomer Materials for Energy, Campus St Jérôme, 13013 Marseille, France; suanto.syahputra@etu.univ-amu.fr (S.S.); emily.bloch@univ-amu.fr (E.B.); luca.pasquini@univ-amu.fr (L.P.); philippe.knauth@univ-amu.fr (P.K.)

**Keywords:** hybrid materials, electrocatalysis, ionic polymer, oxygen reduction reaction

## Abstract

Anion-conducting organic–inorganic polymers (OIPs), constructed using metal–organic framework (MOF)-like structures with non-toxic, non-rare catalytic metals (Fe^3+^, Zr^4+^), have been developed. The incorporation of MOF-like structures imparts porosity to the polymers, classifying them as porous organic polymers (POPs). The combination between catalytic activity, ion conduction, and porosity allows the material to act as one-component catalytic electrodes. A high catalytic activity is expected since the entire surface area contributes to electrocatalysis, rather than being restricted to triple-phase boundaries. The synthesis involved anchoring a synthon onto a commercial polymer, assembling organo-metallic moieties, and functionalizing with quaternary ammonium (QA) groups. Two hybrid materials, Zr-POP-QA and Fe-POP-QA, were thoroughly characterized by NMR, FTIR, XPS, BET surface area (≈200 m^2^/g), and TGA. The resulting electrodes demonstrated a high electrochemically active surface area and a high efficiency for the oxygen reduction reaction (ORR), a critical process for energy storage and conversion technologies. The performance was characterized by a 4-electron reduction pathway, a high onset potential (≈0.9 V vs. RHE), and a low Tafel slope (≈0.06 V). We attribute this efficiency to the high active surface area, which results from the simultaneous presence of catalytic transition metal ions (Zr or Fe) and ion conducting groups.

## 1. Introduction

Organic–inorganic polymers (OIPs), constructed using metal–organic framework (MOF)-like design principles, can achieve high porosity, tailored functionality, and structural stability. However, unlike traditional MOFs, they can have amorphous structures, resulting in materials that retain some of the intrinsic qualities of MOFs (such as high surface area and tuneable functionality) with the versatility, flexibility, and processing advantages of polymers [1].

These OIPs can be considered a subset of porous organic polymers (POPs). POPs are a class of materials characterized by their high surface area, tuneable porosity, and ability to incorporate various functional groups, ideal for a wide range of applications, including catalysis, gas storage, and separation [2,3,4,5]. Unlike traditional MOFs, POPs are constructed from organic building blocks [6,7] and often lack the metal centres that define MOFs. The concept of combining organic monomers with metal–organic coordination in hybrid structures can create materials with peculiar properties, such as porous polymer with the catalytic capabilities of metal ions. Furthermore, by incorporating ion-conducting groups into the structure, these materials gain the ability to transport ions, which is particularly advantageous for electrochemical applications. This synergy leads to materials with increased surface areas and abundant active sites, making them ideal for use as single component electrodes, particularly for the oxygen reduction reaction (ORR).

The ORR is crucial for the functionality and efficiency of fuel cells and metal–air batteries, playing a key role in the overall energy storage and conversion process. In alkaline conditions, where the ORR is less energetically demanding than in acidic media, non-noble metal electrocatalysts, including carbon materials [8,9,10], are available. In general, the electrocatalytic reaction takes place at the triple-phase boundary, the interface where the gas phase, the electrocatalyst, and the ionomer coexist. To optimize this process, adding an anion exchange ionomer (AEI) is essential. The AEI facilitates the removal of hydroxide ions from the electrode; an optimized microstructure with well-distributed catalyst and ionomer particles, which enhances the triple-phase boundary area, improves electrode kinetics and overall efficiency [11].

Embedding transition metals such as Fe, Co, or Ni into a porous polymer can significantly boost the ORR activity [12]. A material that combines electrocatalytically active centres, the cations in the MOF-like component, with a covalently linked hydroxide ion conducting polymer is expected to be particularly active because the whole surface area of the electrode can become catalytically active, not only the limited triple phase boundaries. Consequently, a catalyst layer composed of this single porous ionomer with dual functionality could considerably increase the efficiency of electrocatalytic processes. Cobalt, frequently used for ORR applications [13], is considered a metal with neurological, cardiac, hematological, and endocrine toxicity, which reduces the sustainability of catalysts. Therefore, there is a pressing need to develop catalysts and electrocatalysts that do not rely on toxic or rare metals. MOFs were frequently used as precursors for developing catalytic materials, with several studies reporting their activity for the oxygen reduction and evolution reactions when combined with ion exchange ionomers [14,15,16,17,18]. Similarly, POPs based on various frameworks containing metals, mixed with ion exchange polymers, have also been explored for the ORR [2,12,19,20]. In these examples, the catalytic materials are typically prepared through physical mixing, where the interactions between components rely on hydrogen bonding, π-π interactions, and van der Waals forces. However, such mixtures often suffer from phase separation, poor mechanical properties, limited processability, and low stability under operational conditions [21]. To address these issues, emerging approaches focus on creating hybrid materials that combine covalently linked “hard” MOFs with “soft” polymers. One innovative strategy is the “polymerization of the framework”, where organic ligands serve dual roles as both the building blocks for the MOF and the polymer chain [22,23,24,25,26]. This method results in covalently bonded structures that enhance the material properties, leading to more stable and efficient porous organic polymers. MOFs covalently linked to commercial polymers are reported in different applications, including fuel cells [27], lithium-ion batteries [28], and CO_2_ and H_2_ separation [29,30,31]. In contrast to the extensive studies on proton-conductive MOFs and related membranes [32,33,34], rarely do reports focus on MOFs mixed with AEIs [35,36,37].

In this study, we synthesized hydroxide ion-conducting organic–inorganic polymers covalently linked to Zr- and Fe-MOF-like structures. Polysulfone (PSU) was chosen as the polymer matrix for its versatility in MOF-like anchoring and was further functionalized with quaternary ammonium (QA) groups to add anion conductivity.

The resulting hybrid materials integrate three key functional properties: catalytic centres (Zr^4+^ or Fe^3+^) for enhanced electrocatalytic activity; ion-conducting groups (QA) to facilitate ion transport; porosity introduced by the MOF-like structure to optimize oxygen adsorption and catalytic performance. A key advantage of these materials is that they function as one-component catalytic electrodes, eliminating the need for additional components.

Our synthesis strategy involved first anchoring a benzenedicarboxylate (terephthalate) derivative onto PSU as a MOF precursor, then assembling the MOF-like structure onto the polymer backbone, and finally, introducing quaternary ammonium groups to impart ionic conductivity.

Two types of hybrid materials were synthesized: Zr-POP-QA and Fe-POP-QA. Their electrocatalytic performance was then evaluated for the ORR, demonstrating their potential as efficient ion-conducting catalysts.

## 2. Results and Discussion

**Figure 1:**
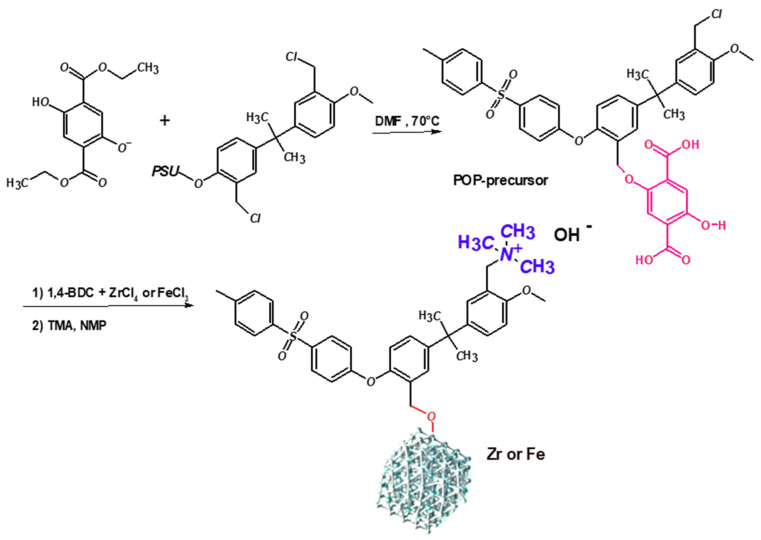
Simplified reaction pathways of Zr-POP-QA and Fe-POP-QA.

The strategy to create porous hybrid ion-conducting polymers involved linking a synthon molecule to the backbone of PSU forming the POP-precursor, creating a site where the metal–organic part can self-assemble. The simplified reaction sequence for the synthesis of Zr- and Fe-POP-QA is illustrated in Figure 1, with a more detailed representation provided in the Supporting Information (Schemes S1–S3).

The ^1^H NMR spectrum of the POP-precursor (Figure S1) confirms the presence of a terephthalic moiety covalently linked to PSU with a degree of functionalization of 0.3.

The organo-metallic moieties were assembled by reaction with additional terephthalic acid via a solvothermal method, adapting procedures previously used in the synthesis of UiO-66 [38] and Fe^3+^-BDC-MOF (analogue of Fe-MIL-53) [39] (Scheme S2). As reported in earlier studies on the synthesis of Fe-MOFs with a bidentate linker, the solvent DMF acts as a ligand in the process [39,40]. The introduction of quaternary ammonium groups, following the procedure in ref. [41], is limited by Manning condensation, which occurs when the distance between adjacent ammonium groups is less than 0.7 nm [42] (Scheme S3).

The presence of the inorganic component reduces the solubility of the polymer, making the NMR analysis of Zr-, and Fe-POP difficult. Furthermore, Fe-POP is a paramagnetic compound that further complexifies the NMR analysis. However, functionalization with quaternary ammonium ions enhances the solubility in polar solvents. The ^1^H NMR spectrum of Zr-POP-QA, shown in Figure S4, confirms the presence of ionic groups and organo-metallic moieties grafted onto the polymer.

The presence of the ammonium groups was also established by potentiometric titration that indicated an ion exchange capacity of 1.93 and 1.04 meq/g for Zr and Fe derivatives.

**Figure 2:**
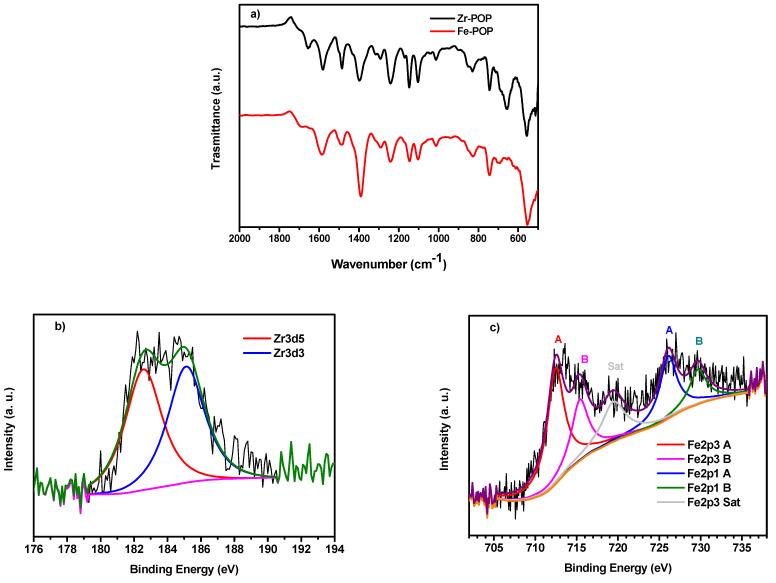
(**a**) FTIR spectra of Zr-POP (black) and Fe-POP (red). XPS spectra of (**b**) Zr-POP-QA and (**c**) Fe-POP-QA.

The FTIR spectra of chloromethylated PSU and POP-precursor are presented in Figure S3 along with the corresponding signal attributions. The presence of the peak at 1730 cm^−1^ (C=O stretch) in the POP-precursor indicates the successful grafting of the terephthalic moiety to the PSU backbone. The FTIR spectra of Zr- and Fe-POPs (Figure 2a) show that the incorporation of Zr- or Fe-derivatives into the PSU matrix significantly modifies the vibrational characteristics of the material, particularly in the carboxylate regions. For the Zr-derivate, carboxylate groups coordinated with the metal centre present two peaks: the asymmetric stretch at 1650 cm^−1^ and the symmetric stretch at 1400 cm^−1^. The coordination with Zr alters the carboxylate double bond due to the resonance effect, reducing the bond order from a pure double bond to approximately 1.5 [43]. In the case of Fe-POPs, Fe interacts with the carboxylate in a tridentate coordination rather than tetradentate, shifting the sym stretch of the carboxylate bond to 1380 cm^−1^. Zr-O (oxide/hydroxide/carboxylate) bonds appear at 750 and 650 cm^−1^ [44], while Fe-O vibrations are observed at 820, 730 and 543 cm^−1^ [45,46]. This analysis, along with the combination of the asym and sym bands, clearly confirms the incorporation of Zr- and Fe-moieties into the polymer. The FTIR spectra of Zr- and Fe-POP-QA are reported in Figure S3b. The characteristic peaks of the aminated compounds (CH_3_ and –N^+^–CH_2_ stretch of quaternary ammonium) overlap with the CH_3_ stretch of the polysulfone backbone and with the benzene mode [47]. In conclusion, the key finding of covalent bonding between the metal–organic framework and anion-conducting polymer is confirmed by FTIR.

The XPS spectrum of Zr-POP-QA in the Zr 3d region (Figure 2b) shows two components at 182.6 (3d_5/2_) and 185.3 eV (3d_3/2_) attributed to Zr-O bonds, indicating the presence of Zr^4+^ linked to oxygen [48]. The percentage of Zr is 1.4 atom%. The C 1s region, reported in Figure S4, reveals a total carbon content of 70.4 atom%. Nitrogen is present as C-N at 399.4 eV with an amount of 5.6 atom%.

Figure 2c presents the XPS spectrum of Fe 2p region. The spectrum shows two different groups of signals corresponding to the spin–orbit doublets of Fe 2p_3/2_ and 2p_1/2_, with satellite peaks observed around 720.0 and 735.0 eV. The deconvolution of the Fe 2p_3/2_ region reveals two components at 712.7 and 715.4 eV. As reported in ref [49] for tris(β-diketonato)iron(III) complexes, these components reflect the presence of various isomers resulting from the interaction between Fe(III) and the ligands. Specifically, when the ligands are unsymmetrical, the binding energy is similar to what we observed in our experiments [49]. The POP-precursor and terephthalic acid, in the presence of iron, can assemble in different configurations, leading to the formation of various isomers. The total amount of iron is only 0.6 atom%, below the quantity of metal in Zr-POP-QA, indicating that the bidentate linker might not have coordinated all iron ions. The C 1s region for this sample, as shown in Figure S5, indicates a total carbon content of 74.6 atom%. Nitrogen is present as C-N at 399.7 eV with a content of 4.0 atom%.

A typical optical micrograph of the Fe-POP-QA electrode on carbon paper is presented in the supporting information (Figure S6). The image clearly shows a relatively homogeneous distribution of the brownish hybrid material on top of the carbon fibres.

**Figure 3:**
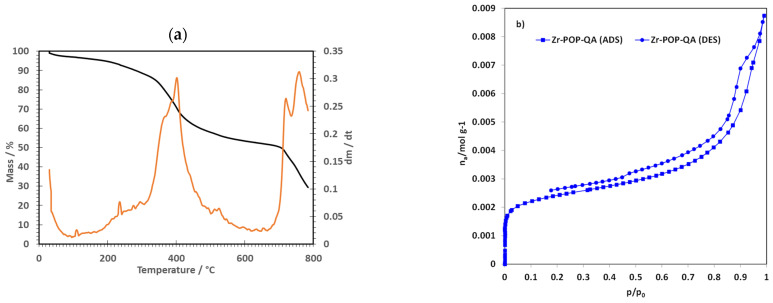
(**a**) Thermogram of Fe-POP-QA under air; (**b**) BET adsorption/desorption isotherm of Zr-POP-QA.

The high-resolution thermogravimetric analysis in Figure 3a displays two key mass losses, indicating different stages of material decomposition. The first one, with a peak at approximately 400 °C, is associated with the decomposition of the “organic part”, i.e., the polymer main chain containing the quaternary ammonium groups but not the metal moieties [50]. The shoulder at 360 °C is attributable to terephthalic acid, as previously reported [51]. The decomposition of the quaternary ammonium groups starts at around 200 °C. The second significant mass loss occurs above 700 °C and represents about 50 wt% of the material. It is associated with the decomposition of the inorganic part covalently linked to the polymer. The TGA thus unambiguously confirms the presence of covalently bonded organic and inorganic parts in the POP.

The surface area of Zr-POP-QA was evaluated by N_2_ sorption measurements at 77 K. The adsorption isotherm shown in Figure 3b is of type 4 [52]. The calculated BET surface area, in the domain p/p° = 0.05–0.15, is quite high: 192.5 m^2^/g. In the high-pressure region above p/p° = 0.8, mesopores are filled, resulting in a hysteresis loop. The average pore diameter, calculated using the BJH method, is 6.3 nm.

The BET surface area of Zr-POP is even higher, around 269 m^2^/g, as detailed in the supporting information (Figure S7). In contrast, the surface area of unmodified PSU is only about 6 m^2^/g [53]. Although a slight reduction is observed after quaternization, the significant increase in polymer surface area after functionalization highlights the effectiveness of this approach to create a porous polymer.

**Figure 4:**
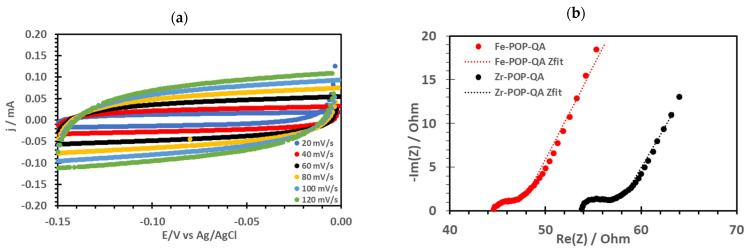
(**a**) Cyclovoltammograms of Fe-POP-QA at various scan rates and (**b**) impedance spectra of POP-QA electrodes in 0.1 M KOH. Dots: experiment, dashed lines: Zfit plot.

The performance of these new materials was explored as electrocatalysts for the ORR. The high surface area of the samples can also be observed by capacitance measurements. Cyclovoltammograms in the non-Faradaic region of a Fe-POP-QA electrode at various scan rates dU/dt are shown in Figure 4a.

The DC capacitances C (Table 1) are obtained according to Equation (1):

j = C dU/dt.(1)

Considering the geometrical electrode area, the electrode capacitances are 2.57 and 4.18 mF/cm^2^ for Fe- and Zr-POP-QA, respectively. A higher electrochemically active surface area (ECSA) is observed for the zirconium than for the iron sample, which is probably related to the higher metal content and the higher IEC of Zr-POP-QA. Assuming a typical double-layer capacitance of 20 μF/cm^2^ and given the electrode mass of 0.22 mg, an ECSA of 95 m^2^/g can be assessed for Zr-POP-QA, which is only slightly lower than the BET value, consistent with a highly electrochemically active electrode surface.

Impedance spectra are presented in Figure 4b. The non-linear least-square fits are based on an equivalent circuit including a resistance R1 corresponding to the sum of the alkaline electrolyte resistance and the porous polymer electrode resistance. In series, a parallel R2-Q2 element contains the charge transfer resistance R2 and the constant phase element Q2, representing the interfacial capacitance. The impedance of a constant phase element Q can be written according to Equation (2):

*Z*(*CPE*) = (*iω*)^−*n*^/*Q*. (2)

Here, *Q* is the CPE parameter, and *n* is the CPE exponent. *i* is the imaginary unit, and *ω* is the angular frequency. The best-fit parameters are reported in Table 1. The higher R1 value of Zr-POP-QA is consistent with its higher electrode resistance. The lower R2 for Fe-POP-QA is in agreement with the larger current densities observed for this sample. The constant phase element values Q3 are consistent with the DC capacitance values. Both impedance and cyclovoltammetric measurements are in agreement with a high electrochemically active surface area of the electrode material.

**Figure 5:**
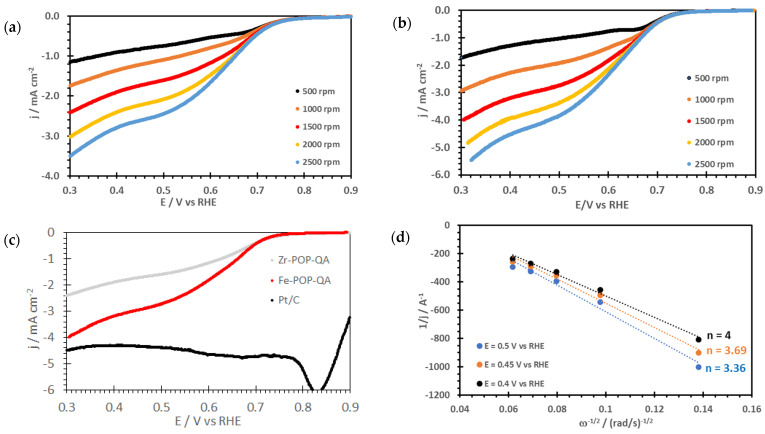
Linear Sweep Voltammograms for the ORR in oxygen-saturated 0.1 M KOH at various RDE speeds: (**a**) Zr-POP-QA, (**b**) Fe-POP-QA, and (**c**) comparison with benchmark Pt/C cloth at 1500 rpm. (**d**) Koutecky–Levich plots for Fe-POP-QA.

The linear sweep voltammograms for the ORR at various RDE speeds are reported in Figure 5a,b. One notices a high electrocatalytic performance, especially for the Fe-POP-QA electrodes with about 60% higher cathodic current at 0.4 V vs. RHE than Zr-POP-QA, although the amount of iron is significantly lower than that of zirconium. A comparison with the benchmark Pt/C cloth is shown in Figure 5c. Previously, Zr-UiO-66-NO_2_ made using nitroterephthalic acid showed excellent oxygen adsorption properties; when combined with Co-phthalocyanine and carbon nanotubes (Co-CNT), it was shown to be an outstanding electrocatalyst for the ORR [54]. In our study, the Zr-UiO-66-like structure, assuring the oxygen adsorption as necessary first step of the ORR, with a covalently linked anion conducting polymer enhancing the mass transport kinetics of the hydroxide ions, proved to be efficient for the ORR alone, even without the Co-CNT part. One can argue that the presence of the ion-conducting part compensates partly the absence of the Co-doped CNT. Fe-MIL-53 with a covalently grafted Ni^2+^-(2-pyridine carboxaldehyde) complex was described as an effective electrocatalyst for the OER, attributed to the presence of the Ni complex, and with a high stability in alkaline medium [55]. In our study, Fe-POP-QA without nickel complex, but with a covalently linked hydroxide-conducting polymer, showed interesting electrocatalytic activity also for the ORR. One can conclude that the synergy of oxygen adsorption in the Fe-MOF-like part, combined with the charge transfer step taking place on Fe^3+^ and hydroxide ion conduction in the ionomeric part, support promising electrocatalytic activity even without Ni complex.

The number of exchanged electrons can be assessed using the Koutecky–Levich Equation (3):

(3) $$\frac{1}{i}=\frac{1}{i^{k}}+\frac{1}{B\cdot \omega^{\frac{1}{2}}}.$$

In this relation, the limiting current *i* is related to the kinetic current *i^k^* and the angular frequency *ω* (in rad s^−1^) [56,57]. *B* is the Levich constant:

(4) $$B=0.62\cdot A\cdot n\cdot F\cdot c\left(O_{2}\right)\cdot {D\left(O_{2}\right)}^{\frac{2}{3}}\cdot v^{-\frac{1}{6}}.$$

In Equation (4), *A* is the geometrical electrode area, *n* the number of exchanged electrons, and *F* Faraday’s constant (96,485 C mol^−1^). *c*(*O*_2_) is the oxygen concentration (1.2 × 10^−6^ mol cm^−3^ [58,59,60]) and *D*(*O*_2_) the oxygen diffusion coefficient in oxygen-saturated 0.1 M KOH (1.9 × 10^−5^ cm^2^ s^−1^ [58,60]). *ν* is the kinematic viscosity of a 0.1 M KOH solution (8.7 × 10^−3^ cm^2^ s^−1^) [60,61].

Koutecky–Levich plots (Figure 5d) show that a large amount of 4-electron reduction is observed; the number of exchanged electrons attains n = 4 at E = 0.4 V vs. RHE for Fe-POP-QA, whereas n = 3.12 for Zr-POP-QA. The higher electrocatalytic activity of the Fe-containing electrode was expected given the relatively easy valence change in Fe ions, compared to Zr ions. However, even the Zr-POP-QA shows a significant electrocatalytic performance, which can be attributed to the high ECSA with catalytically active centres and ionic groups in nanometric proximity.

The low Tafel slopes (RT/nαF) reported in Table 2 confirm a good electrocatalytic activity and indicate a two-electron transfer process as rate-limiting step for both electrodes consistent with the predominant 4-electron reduction pathway.

Table 2 highlights the effectiveness of the electrocatalysts. A comparison with existing literature about catalysts based on Co-free POPs shows that the ORR performance of our samples is excellent, especially because they do not rely on rare or toxic metals. Furthermore, they are anion conducting, which contributes to their high performance, and is advantageous because they do not require additional components.

**Figure 6:**
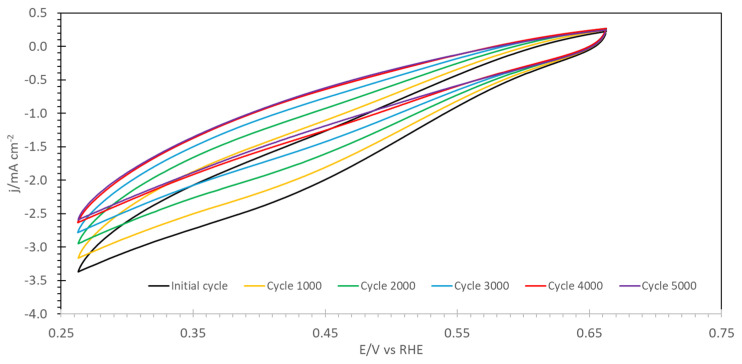
Accelerated degradation tests for Zr-POP-QA: cyclovoltammetric curves for the ORR in oxygen-saturated 0.1 M KOH solution at 1500 rpm RDE speed as function of the cycle number.

Finally, accelerated degradation tests were performed. The evolution of cyclovoltammograms for the ORR during 5000 cycles is shown in Figure 6 and Figure S8. One recognizes, for both samples, a decrease in the current by about 20% (Zr) or 25% (Fe) over 5000 cycles. The largest decrease is seen in the mass-transport dominated region below 0.55 V vs. RHE.

The FTIR spectra for the sample Zr-POP-QA before and after the accelerated degradation test are presented in Figure S9. The spectrum after the test is dominated by the presence of water especially at 1630 cm^−1^ (H_2_O bending) [66] related to the long immersion in KOH solution. The original peaks are present as shoulders.

Impedance spectra recorded before and after the accelerated test (Figure S10 and Table S1) show a slight increase in the electrode and charge transfer resistances, indicating a decrease in the electrocatalytic activity. A second similar test over 5000 cycles gave consistent results. One can conclude that the stability of the POP-QA electrocatalysts is quite good, underlining the potential of these materials.

## 3. Materials and Methods

### 3.1. Materials

Polysulfone (PSU) UDEL P-1800 NT11, diethyl 2,5-dihydroxyterephthalate 97%, terephthalic acid 98% (1,4-BDC), ZrCl_4_ 99.5%, FeCl_3_ 97%, trimethylamine (TMA, 4.2 M in ethanol), *N*,*N*-dimethylformamide (DMF), dimethyl sulfoxide (DMSO), 1-methyl-2-pyrrolidone (NMP), and other chemicals were used as received from Sigma-Aldrich (Milano, Italy). Carbon paper (AvCarb EP55) and Pt/C 60% cloth gas diffusion electrode (GDE, 0.5 mg/cm^2^) were purchased from Fuel Cell Store.

### 3.2. Synthesis

#### 3.2.1. POP-Precursor

The POP-precursor was synthesized from chloromethylated polysulfone (PSU-CH_2_Cl) following the procedure described in the literature [41,67,68]. Two different solutions were prepared. In the first, 1 g (1.9 mmol) of PSU-CH_2_Cl (degree of chloromethylation, DCM 1.7) was dissolved in 25 mL of anhydrous DMF under N_2_ flux at 50 °C, and then 0.05 g of KI (0.32 mmol) was added under stirring. In the second solution, 0.41 g (1.6 mmol) of diethyl 2,5-dihydroxyterephthalate (in molar ratio 0.5 with respect to DCM) was dissolved in 10 mL of anhydrous DMF and then K_2_CO_3_ (0.22 g, 1.6 mmol) was added. This mixture was heated at 80 °C and maintained overnight under N_2_ flux. The two solutions were combined and left to react for 3 days at 70 °C. The hydrolysis of the residual esters was carried out with a 10 wt% solution of NaOH in 50:50 H_2_O/MeOH at 60 °C for 2 h. The solution was finally precipitated with 15 mL of 2 M HCl and 15 mL of EtOH to obtain the H-form and digested overnight. The filtered POP-precursor was washed several times with EtOH and water and then stored on P_2_O_5_. The yield was 75%.

^1^H NMR (DMSO-*d*_6_): δ = 1.6 ((CH_3_)_2_-PSU, 6H), δ = 4.4 (PSU-CH_2_-Cl, 2.3 H), δ = 5.30–5.42 (PSU-CH_2_-O, 0.6 H), δ = 6.7–8.1 (PSU aromatic region), δ = 9.6 (Ph-OH, 0.3 H). The degree of functionalization, measured by comparison between the area of PSU methyl groups ((CH_3_)_2_-PSU) and the ether linkage (PSU-CH_2_-O), was 0.3. The ^1^H NMR spectrum is reported in Figure S1.

#### 3.2.2. Hybrid Organic–Inorganic Polymers (Zr- and Fe-POP)

*Solution 1.* A total of 0.50 g (0.87 mmol) of POP-precursor was dissolved in 20 mL of anhydrous DMF under N_2_ flux and then 0.125 g (0.75 mmol) of terephthalic acid was added and mixed under stirring for 30 min.

*Solution 2.* A total of 0.20 g of ZrCl_4_ (0.75 mmol) or 0.24 g of FeCl_3_ (1.5 mmol) was dissolved in 5 mL of anhydrous DMF, followed by the addition of 0.05 mL of double distilled water and stirred for 30 min.

The solution 2 was added to the solution 1, mixed for 30 min, poured inside a Teflon bottle and inserted in an oven for 16 h at 120 °C. The resulting product was washed in 0.1 M HCl solution and acetone (3 times) and stored under P_2_O_5_. The yields were 100% for Zr-POP and 50% for Fe-POP.

#### 3.2.3. Quaternization of Zr- and Fe-POP

Zr-POP-QA

0.40 g (0.60 mmol) of Zr-POP was dissolved in NMP, then 0.24 mL of TMA (1 mmol) was added and left for 3 days at 80 °C under stirring.

Fe-POP-QA

0.10 g (0.15 mmol) of Fe-POP was dissolved in 15 mL of DMSO, then 0.06 mL of TMA (0.25 mmol) was added and left for 3 days at 80 °C.

Both products were dried under high vacuum, washed with H_2_O and dried again.

^1^H NMR (Zr-POP-QA, DMSO-*d*_6_): δ = 1.6 ((CH_3_)_2_-PSU, 6H), δ = 2.8–3.0 (-N^+^(CH_3_)_3_, 11.6 H), δ = 4.2–4.6 (PSU-CH_2_-N^+^(CH_3_)_3_, 2.3 H), δ = 5.5–6.0 (PSU-CH_2_-O, 0.6 H), δ = 6.7–8.1 (PSU aromatic region). The degree of amination, measured by comparison between the area of CH_3_-PSU and PSU-CH_2_-N^+^(CH_3_)_3_, was 1.1. The ^1^H NMR spectrum is reported in Figure S2.

#### 3.2.4. Electrode Fabrication

POP-QA inks were prepared dispersing 25 mg of sample in 280 μL of solvent. A mixture of DMSO (224 μL) and isopropyl alcohol (56 μL) was used for Zr-POP-QA and toluene was used for Fe-POP-QA.

In both cases, the mixture was stirred at room temperature for one night and then sonicated for 1 h. A total of 2.5 μL of the ink were deposited by drop-casting on an acid-modified carbon paper [9], and were carefully dried under vacuum by a rotary pump at 40 °C for about 4 h. The amount of electrocatalyst was thus 0.22 mg.

### 3.3. Characterization Techniques

#### 3.3.1. Ion Exchange Capacity

The IEC (milliequivalents per gram of dry polymer) was determined by potentiometric acid–base titration. The fine powders (Zr- and Fe-POP-QA) were treated with 0.1 M NaOH solution for 2 days to have the OH^-^ form and washed in bidistilled water for 2 days to remove excess base. After drying over P_2_O_5_ for 72 h, samples were weighed and immersed in a 0.02 M HCl. The acid solution was then back-titrated with 0.02 M NaOH.

#### 3.3.2. ^1^H NMR Spectroscopy

^1^H NMR spectra were recorded with a Bruker Avance 700 (Bruker, Milano, Italy) spectrometer operating at 700.18 MHz using DMSO-*d*_6_.

#### 3.3.3. FTIR Spectroscopy

FTIR spectra were recorded in transmission mode in the range of 4000–500 cm^−1^ using a Perkin Elmer Spectrum (Perkin Elmer, Milano, Italy) 2 IR spectrometer equipped with an ATR Zinc Selenide (ZnSe) crystal.

#### 3.3.4. X-Ray Powder Diffraction (XRD)

XRD patterns were collected using a Panalytical X’Pert PRO diffractometer with CuKα radiation, a step size of 0.033°, and a step scan of 50 s. The XRD diffractograms show an amorphous pattern for both samples.

#### 3.3.5. X-Ray Photoelectron Spectroscopy (XPS)

XPS analyses were carried out by using an Escalab MkII (Vacuum Generators Ltd., St. Leonards, UK) spectrometer with non-monochromatic Al Kα (1486.6 eV) source. The powder samples were pressed on pure Au (99.99%) foil. The binding energy (BE) scale was corrected by positioning the C 1s peak of aliphatic carbon at BE = 285.0 eV and controlling the position of the Fermi level at BE = 0 eV.

#### 3.3.6. Thermogravimetric Analysis (TGA)

The high-resolution TGA was performed between 30 and 800 °C with a maximum heating rate of 3 K/min under air flow in Pt sample pans; a TA Q500 apparatus (TA instruments, New Castle, DE, USA) was used.

#### 3.3.7. Brunauer–Emmett–Teller (BET) Analysis

The BET surface area, total pore volume, and mean pore diameter were determined via nitrogen gas sorption at 77 K. Prior to adsorption, samples were vacuum degassed at 250 °C overnight.

#### 3.3.8. Electrochemical Measurements

The used three-electrode cell included a rotating disc electrode (RDE, 0.28 cm^2^ area, OrigaTrod, OrigaLys, Rillieux-la-Pape, France), a 4 cm^2^ Pt counter-electrode, and an Ag/AgCl reference electrode (E = 0.197 V vs. SHE). Ohmic drop correction was applied and all potentials were expressed vs. the reversible hydrogen electrode (RHE). The electrolyte was oxygen-saturated 0.1 M KOH.

Cyclic voltammetry (CV), linear sweep voltammetry (LSV), chronoamperometry (CA), and electrochemical impedance spectroscopy (EIS) were applied at ambient temperature using a Biologic VMP3 potentiostat. The scan rates were 20–120 mV/s (CV) and 5 mV/s (LSV). The rotating speed of the RDE was varied between 500 and 2500 rpm. The impedance spectra were recorded with an a.c. amplitude of 20 mV between 1 Hz and 1 MHz.

## 4. Conclusions

The synthesis of hybrid organic–inorganic polymers (OIPs) with anion-conducting groups, a BET surface area of approximately 200 m^2^/g and non-toxic, non-rare metals (Zr or Fe) integrated into MOF-like structures was successfully achieved. The use of covalent linkages between the metal–organic component and the polymer matrix addresses issues of phase separation that often hinder the performance of other hybrid materials. Two materials, Zr-POP-QA and Fe-POP-QA, were employed as single-component catalytic electrodes for the oxygen reduction reaction (ORR). The results demonstrated a high electrochemically active surface area, significant 4-electron reduction, a high onset potential (≈0.9 V vs. RHE) and low Tafel slope (≈0.06 V). Fe-POP-QA, which contains less metal (0.6 at%), shows a higher limiting current density and can be considered even more promising than Zr-POP-QA. The high electrocatalytic activity is attributed to the large electrochemically active surface area, which is enhanced by the simultaneous presence of catalytically active metal ions (Zr or Fe) and effective hydroxide ion conduction. Accelerated degradation tests for both materials using cyclovoltammetry (5000 cycles) show a low decrease in the electrocatalytic activity by about 20%. Overall, the reported synthesis represents a significant step forward in developing advanced materials with tailored properties for energy and catalysis applications.

## Figures and Tables

**Table 1:** DC capacitances C and non-linear best-fit impedance parameters of POP-QA electrodes.

Sample	C /µF	R1 /Ω	R2 /Ω	Q2 /µFs ^n−1^	n2	Q3 /µFs ^n−1^	n3
Fe-POP-QA	728	44.6	2.7	42.4	0.82	1240	0.72
Zr-POP-QA	1170	53.6	3.8	54.0	0.74	1990	0.69

**Table 2:** Comparison with Co-free electrodes from literature for the ORR: onset potentials E_on_ and half-wave potentials E_1/2_ vs. RHE, number of exchanged electrodes n at E vs. RHE = 0.4–0.6 V, and Tafel slopes b in oxygen-saturated 0.1 M KOH.

Sample	E_on_ /V	E_1/2_ /V	n	b /mV	Ref.
Fe-POP-QA	0.90	0.67	4.0	68	This work
Zr-POP-QA	0.90	0.68	3.2	60	This work
Conjugated microporous polymers	0.82	-	4.0	-	[62]
Bola-amphiphilic conjugated microporous polymers	0.81	-	3.8–3.9	-	[63]
Phthalocyanine-porphyrin-based conjugated microporous polymers	0.93	0.86	4.0	33	[64]
Corrole-based POP	0.81	0.66	3.0	90	[65]

## Data Availability

No data were used for the research described in this article.

## Supplementary Materials

The following supporting information can be downloaded at: https://www.mdpi.com/article/10.3390/molecules30061230/s1.

## References

[1] Kalaj M., Bentz K.C., Ayala S., Palomba J.M., Barcus K.S., Katayama Y., Cohen S.M. (2020). MOF-Polymer Hybrid Materials: From Simple Composites to Tailored Architectures. Chem. Rev..

[2] Yang D.-H., Tao Y., Ding X., Han B.-H. (2022). Porous organic polymers for electrocatalysis. Chem. Soc. Rev..

[3] Zhang T., Xing G., Chen W., Chen L. (2020). Porous organic polymers: A promising platform for efficient photocatalysis. Mater. Chem. Front..

[4] Mohamed M.G., El-Mahdy A.F.M., Kotp M.G., Kuo S.-W. (2022). Advances in porous organic polymers: Syntheses, structures, and diverse applications. Mater. Adv..

[5] Naz N., Manzoor M.H., Naqvi S.M.G., Ehsan U., Aslam M., Verpoort F. (2024). Porous organic polymers; an emerging material applied in energy, environmental and biomedical applications. Appl. Mater. Today.

[6] Song W., Zhang Y., Tran C.H., Choi H.K., Yu D.-G., Kim I. (2023). Porous organic polymers with defined morphologies: Synthesis, assembly, and emerging applications. Prog. Polym. Sci..

[7] Chen W., Chen P., Zhang G., Xing G., Feng Y., Yang Y.-W., Chen L. (2021). Macrocycle-derived hierarchical porous organic polymers: Synthesis and applications. Chem. Soc. Rev..

[8] Nallayagari A.R., Sgreccia E., Pasquini L., Sette M., Knauth P., Di Vona M.L. (2022). Impact of Anion Exchange Ionomers on the Electrocatalytic Performance for the Oxygen Reduction Reaction of B-N Co-doped Carbon Quantum Dots on Activated Carbon. ACS Appl. Mater. Interfaces.

[9] Nallayagari A.R., Sgreccia E., Pasquini L., Vacandio F., Kaciulis S., Di Vona M.L., Knauth P. (2022). Catalytic electrodes for the oxygen reduction reaction based on co-doped (B-N, Si-N, S-N) carbon quantum dots and anion exchange ionomer. Electrochim. Acta.

[10] Knauth P., Sgreccia E., Nallayagari A.R., Pasquini L., Narducci R., Di Vona M.L. (2023). Electrocatalytic composites of carbon quantum dots and anion exchange ionomers for the oxygen reduction reaction. Eur. J. Mater..

[11] Jinnouchi R., Kudo K., Kodama K., Kitano N., Suzuki T., Minami S., Shinozaki K., Hasegawa N., Shinohara A. (2021). The role of oxygen-permeable ionomer for polymer electrolyte fuel cells. Nat. Commun..

[12] Li H., Pan F., Qin C., Wang T., Chen K.-J. (2023). Porous Organic Polymers-Based Single-Atom Catalysts for Sustainable Energy-Related Electrocatalysis. Adv. Energy Mater..

[13] Park H., Oh S., Lee S., Choi S., Oh M. (2019). Cobalt- and nitrogen-codoped porous carbon catalyst made from core–shell type hybrid metal–organic framework (ZIF-L@ZIF-67) and its efficient oxygen reduction reaction (ORR) activity. Appl. Catal. B Environ..

[14] Jahan M., Bao Q., Loh K.P. (2012). Electrocatalytically Active Graphene–Porphyrin MOF Composite for Oxygen Reduction Reaction. J. Am. Chem. Soc..

[15] Xia B.Y., Yan Y., Li N., Wu H.B., Lou X.W., Wang X. (2016). A metal–organic framework-derived bifunctional oxygen electrocatalyst. Nat. Energy.

[16] Wang H.-F., Chen L., Pang H., Kaskel S., Xu Q. (2020). MOF-derived electrocatalysts for oxygen reduction, oxygen evolution and hydrogen evolution reactions. Chem. Soc. Rev..

[17] Mártire A.P., Segovia G.M., Azzaroni O., Rafti M., Marmisollé W. (2019). Layer-by-layer integration of conducting polymers and metal organic frameworks onto electrode surfaces: Enhancement of the oxygen reduction reaction through electrocatalytic nanoarchitectonics. Mol. Syst. Des. Eng..

[18] Guo J.N., Lin C.Y., Xia Z.H., Xiang Z.H. (2018). A Pyrolysis-Free Covalent Organic Polymer for Oxygen Reduction. Angew. Chem.-Int. Ed..

[19] Zhou B., Liu L., Yang Z., Li X., Wen Z., Chen L. (2019). Porous Organic Polymer Gel Derived Electrocatalysts for Efficient Oxygen Reduction. ChemElectroChem.

[20] Zhou B.L., Liu L.Z., Cai P.W., Zeng G., Li X.Q., Wen Z.H., Chen L. (2017). Ferrocene-based porous organic polymer derived high-performance electrocatalysts for oxygen reduction. J. Mater. Chem. A.

[21] Feng L., Wang K.-Y., Day G.S., Ryder M.R., Zhou H.-C. (2020). Destruction of Metal–Organic Frameworks: Positive and Negative Aspects of Stability and Lability. Chem. Rev..

[22] Lee J., Lee J., Kim J.Y., Kim M. (2023). Covalent connections between metal–organic frameworks and polymers including covalent organic frameworks. Chem. Soc. Rev..

[23] Zhang Z., Nguyen H.T., Miller S.A., Cohen S.M. (2015). polyMOFs: A Class of Interconvertible Polymer-Metal-Organic-Framework Hybrid Materials. Angew. Chem. Int. Ed. Engl..

[24] Ayala S., Zhang Z., Cohen S.M. (2017). Hierarchical structure and porosity in UiO-66 polyMOFs. Chem. Commun..

[25] MacLeod M.J., Johnson J.A. (2017). Block co-polyMOFs: Assembly of polymer–polyMOF hybrids via iterative exponential growth and “click” chemistry. Polym. Chem..

[26] Gu Y., Huang M., Zhang W., Pearson M.A., Johnson J.A. (2019). PolyMOF Nanoparticles: Dual Roles of a Multivalent polyMOF Ligand in Size Control and Surface Functionalization. Angew. Chem. Int. Ed..

[27] Escorihuela J., Narducci R., Compañ V., Costantino F. (2019). Proton Conductivity of Composite Polyelectrolyte Membranes with Metal-Organic Frameworks for Fuel Cell Applications. Adv. Mater. Interfaces.

[28] Wang Z., Wang S., Wang A., Liu X., Chen J., Zeng Q., Zhang L., Liu W., Zhang L. (2018). Covalently linked metal–organic framework (MOF)-polymer all-solid-state electrolyte membranes for room temperature high performance lithium batteries. J. Mater. Chem. A.

[29] Lin R., Ge L., Hou L., Strounina E., Rudolph V., Zhu Z. (2014). Mixed Matrix Membranes with Strengthened MOFs/Polymer Interfacial Interaction and Improved Membrane Performance. ACS Appl. Mater. Interfaces.

[30] Tien-Binh N., Rodrigue D., Kaliaguine S. (2018). In-situ cross interface linking of PIM-1 polymer and UiO-66-NH2 for outstanding gas separation and physical aging control. J. Membr. Sci..

[31] Wang Z., Tian Y., Fang W., Shrestha B.B., Huang M., Jin J. (2021). Constructing Strong Interfacial Interactions under Mild Conditions in MOF-Incorporated Mixed Matrix Membranes for Gas Separation. ACS Appl. Mater. Interfaces.

[32] Xin Q., Liu T., Li Z., Wang S., Li Y., Li Z., Ouyang J., Jiang Z., Wu H. (2015). Mixed matrix membranes composed of sulfonated poly(ether ether ketone) and a sulfonated metal–organic framework for gas separation. J. Membr. Sci..

[33] Li Z., He G., Zhao Y., Cao Y., Wu H., Li Y., Jiang Z. (2014). Enhanced proton conductivity of proton exchange membranes by incorporating sulfonated metal-organic frameworks. J. Power Sources.

[34] Zhang B., Cao Y., Li Z., Wu H., Yin Y., Cao L., He X., Jiang Z. (2017). Proton exchange nanohybrid membranes with high phosphotungstic acid loading within metal-organic frameworks for PEMFC applications. Electrochim. Acta.

[35] Narducci R., Sgreccia E., Knauth P., Di Vona M.L. (2021). Anion Exchange Membranes with 1D, 2D and 3D Fillers: A Review. Polymers.

[36] Gao L., Li C.-Y.V., Chan K.-Y., Chen Z.-N. (2014). Metal–Organic Framework Threaded with Aminated Polymer Formed in Situ for Fast and Reversible Ion Exchange. J. Am. Chem. Soc..

[37] He X., Gang M., Li Z., He G., Yin Y., Cao L., Zhang B., Wu H., Jiang Z. (2017). Highly conductive and robust composite anion exchange membranes by incorporating quaternized MIL-101(Cr). Sci. Bull..

[38] Donnadio A., Narducci R., Casciola M., Marmottini F., D’Amato R., Jazestani M., Chiniforoshan H., Costantino F. (2017). Mixed Membrane Matrices Based on Nafion/UiO-66/SO3H-UiO-66 Nano-MOFs: Revealing the Effect of Crystal Size, Sulfonation, and Filler Loading on the Mechanical and Conductivity Properties. ACS Appl. Mater. Interfaces.

[39] Ajpi C., Leiva N., Lundblad A., Lindbergh G., Cabrera S. (2023). Synthesis and spectroscopic characterization of Fe^3+^-BDC metal organic framework as material for lithium ion batteries. J. Mol. Struct..

[40] Scherb C., Schödel A., Bein T. (2008). Directing the structure of metal-organic frameworks by oriented surface growth on an organic monolayer. Angew. Chem.-Int. Ed..

[41] Di Vona M.L., Narducci R., Pasquini L., Pelzer K., Knauth P. (2014). Anion-conducting ionomers: Study of type of functionalizing amine and macromolecular cross-linking. Int. J. Hydrogen Energy.

[42] Manning G.S. (1979). Counterion binding in polyelectrolyte theory. Acc. Chem. Res..

[43] Smith B.C. (2018). The Carbonyl Group, Part V: Carboxylates—Coming Clean. Spectroscopy.

[44] Pascual-Colino J., Artetxe B., Beobide G., Castillo O., Fidalgo-Mayo M.L., Isla-López A., Luque A., Mena-Gutiérrez S., Pérez-Yáñez S. (2022). The Chemistry of Zirconium/Carboxylate Clustering Process: Acidic Conditions to Promote Carboxylate-Unsaturated Octahedral Hexamers and Pentanuclear Species. Inorg. Chem..

[45] Namduri H., Nasrazadani S. (2008). Quantitative analysis of iron oxides using Fourier transform infrared spectrophotometry. Corros. Sci..

[46] Parak S., Nikseresht A., Alikarami M., Ghasemi S. (2022). RSM optimization of biodiesel production by a novel composite of Fe(ΙΙΙ)-based MOF and phosphomolybdic acid. Res. Chem. Intermed..

[47] Zhou J., Unlu M., Vega J.A., Kohl P.A. (2009). Anionic polysulfone ionomers and membranes containing fluorenyl groups for anionic fuel cells. J. Power Sources.

[48] Solís R.R., Peñas-Garzón M., Belver C., Rodriguez J.J., Bedia J. (2022). Highly stable UiO-66-NH2 by the microwave-assisted synthesis for solar photocatalytic water treatment. J. Environ. Chem. Eng..

[49] Conradie J., Erasmus E. (2016). XPS Fe 2p peaks from iron tris(β-diketonates): Electronic effect of the β-diketonato ligand. Polyhedron.

[50] Derbali Z., Fahs A., Chailan J.F., Ferrari I.V., Di Vona M.L., Knauth P. (2017). Composite anion exchange membranes with functionalized hydrophilic or hydrophobic titanium dioxide. Int. J. Hydrogen Energy.

[51] Navarathna C.M., Dewage N.B., Karunanayake A.G., Farmer E.L., Perez F., Hassan E., Mlsna T.E., Pittman C.U. (2020). Rhodamine B Adsorptive Removal and Photocatalytic Degradation on MIL-53-Fe MOF/Magnetic Magnetite/Biochar Composites. J. Inorg. Organomet. Polym. Mater..

[52] Thommes M., Kaneko K., Neimark A.V., Olivier J.P., Rodriguez-Reinoso F., Rouquerol J., Sing K.S.W. (2015). Physisorption of gases, with special reference to the evaluation of surface area and pore size distribution (IUPAC Technical Report). Pure Appl. Chem..

[53] Virtanen T., Rudolph G., Lopatina A., Al-Rudainy B., Schagerlöf H., Puro L., Kallioinen M., Lipnizki F. (2020). Analysis of membrane fouling by Brunauer-Emmet-Teller nitrogen adsorption/desorption technique. Sci. Rep..

[54] Zeng S., Lyu F., Sun L., Zhan Y., Ma F.-X., Lu J., Li Y.Y. (2019). UiO-66-NO2 as an Oxygen “Pump” for Enhancing Oxygen Reduction Reaction Performance. Chem. Mater..

[55] Wang Y., Zhou Z., Lin Y., Zhang Y., Bi P., Jing Q., Luo Y., Sun Z., Liao J., Gao Z. (2023). Molecular engineering of Fe-MIL-53 electrocatalyst for effective oxygen evolution reaction. Chem. Eng. J..

[56] Treimer S., Tang A., Johnson D.C. (2002). A consideration of the application of Koutecky-Levich plots in the diagnoses of charge-transfer mechanisms at rotated disk electrodes. Electroanalysis.

[57] Wiberg G.K.H., Zana A. (2016). Levich Analysis and the Apparent Potential Dependency of the Levich B Factor. Anal. Lett..

[58] Davis R.E., Horvath G.L., Tobias C.W. (1967). The solubility and diffusion coefficient of oxygen in potassium hydroxide solutions. Electrochim. Acta.

[59] Yan W.Y., Zheng S.L., Jin W., Peng Z., Wang S.N., Du H., Zhang Y. (2015). The influence of KOH concentration, oxygen partial pressure and temperature on the oxygen reduction reaction at Pt electrodes. J. Electroanal. Chem..

[60] Campos-Roldan C.A., Gonzalez-Huerta R.G., Alonso-Vante N. (2018). Experimental Protocol for HOR and ORR in Alkaline Electrochemical Measurements. J. Electrochem. Soc..

[61] Sipos P.M., Hefter G., May P.M. (2000). Viscosities and densities of highly concentrated aqueous MOH solutions (M+ = Na+, K+, Li+, Cs+, (CH3)(4)N+) at 25.0 degrees C. J. Chem. Eng. Data.

[62] Roy S., Bandyopadhyay A., Das M., Ray P.P., Pati S.K., Maji T.K. (2018). Redox-active and semi-conducting donor-acceptor conjugated microporous polymers as metal-free ORR catalysts. J. Mater. Chem. A.

[63] Singh A., Verma P., Samanta D., Singh T., Maji T.K. (2020). Bimodal Heterogeneous Functionality in Redox-Active Conjugated Microporous Polymer toward Electrocatalytic Oxygen Reduction and Photocatalytic Hydrogen Evolution. Chem.—A Eur. J..

[64] Liu W.B., Wang K., Wang C.M., Liu W.P., Pan H.H., Xiang Y.J., Qi D.D., Jiang J.Z. (2018). Mixed phthalocyanine-porphyrin-based conjugated microporous polymers towards unveiling the activity origin of Fe-N_4_ catalysts for the oxygen reduction reaction. J. Mater. Chem. A.

[65] Bai J., Li R.C., Huang J.C., Shang X.F., Wang G., Chao S.J. (2024). Metal-free corrole-based donor-acceptor porous organic polymers as efficient bifunctional catalysts for hydrogen evolution and oxygen reduction reactions. Inorg. Chem. Front..

[66] Mojet B.L., Ebbesen S.D., Lefferts L. (2010). Light at the interface: The potential of attenuated total reflection infrared spectroscopy for understanding heterogeneous catalysis in water. Chem. Soc. Rev..

[67] Di Vona M.L., Casciola M., Donnadio A., Nocchetti M., Pasquini L., Narducci R., Knauth P. (2017). Anionic conducting composite membranes based on aromatic polymer and layered double hydroxides. Int. J. Hydrogen Energy.

[68] Narducci R., Chailan J.F., Fahs A., Pasquini L., Di Vona M.L., Knauth P. (2016). Mechanical Properties of Anion Exchange Membranes by Combination of Tensile Stress-Strain Tests and Dynamic Mechanical Analysis. J. Polym. Sci. Part B-Polym. Phys..

