# Supplementary material for: One-Component Catalytic Electrodes from Metal–Organic Frameworks Covalently Linked to an Anion Exchange Ionomer
Source: Molecules. 2025 Mar 10;30(6):1230. doi: 10.3390/molecules30061230 (PMC11944300; doi:10.3390/molecules30061230)
Supplement: Supplementary file 1 [file molecules-30-01230-s001.zip › molecules-3498455-supplementary.pdf]

Supporting Information

One-Component Catalytic Electrodes from Metal-Organic Frameworks Covalently Linked to an Anion Exchange Ionomer

R. Narducci[1,*], E. Sgreccia[1], A.V. Montella[1], G. Ercolani[2], S. Kaciulis[3], S. Syahputra[4], E. Bloch[4], L. Pasquini[4], P. Knauth[4], M.L. Di Vona[1,*]

[1] Tor Vergata University of Rome, Dep. Industrial Engineering and International Laboratory: Ionomer Materials for Energy, 00133 Roma, Italy

[2] Tor Vergata University of Rome, Chemistry Department, Via della Ricerca Scientifica, 00133 Roma, Italy

[3] Institute for the Study of Nanostructured Materials, ISMN-CNR, 00015 Monterotondo Stazione, Roma, Italy

[4] Aix Marseille Univ, CNRS, MADIREL (UMR 7246) and International Laboratory: Ionomer Materials for Energy, Campus St Jérôme, 13013 Marseille, France

Synthesis

The reaction sequence started with the deprotonation of phenolic groups. The ester moiety was used to protect the carboxylic acid. The second step was a bimolecular nucleophilic substitution (S_N2) of the phenolate compound on the chloromethyl group of PSU. The sequence ended with the hydrolysis of ester moieties to restore the carboxylic groups (Scheme S1). The reaction's stoichiometry was chosen so that only a fraction of the chloromethyl groups could react with the phenolate compound, leaving the remaining ones available for the successive quaternization reaction.

The ^{1}H NMR spectrum of the precursor is shown in Figure S1.

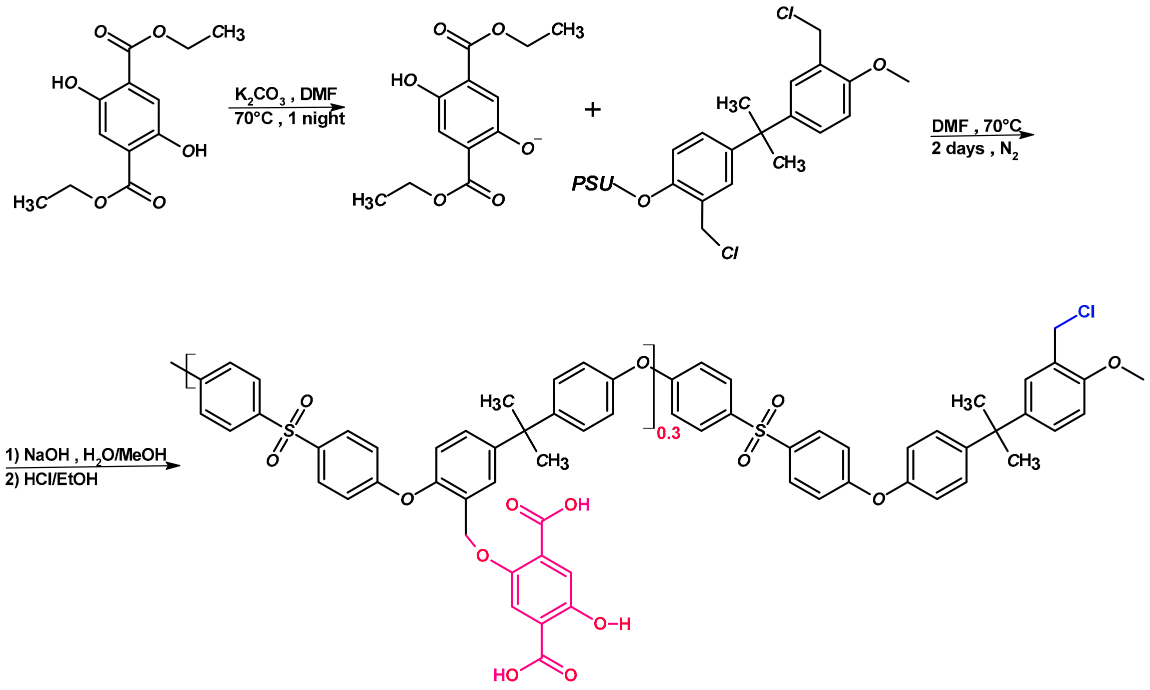

Scheme S1. Reaction sequence of the synthesis of POP-precursor. The index of the repeat unit is estimated by NMR.

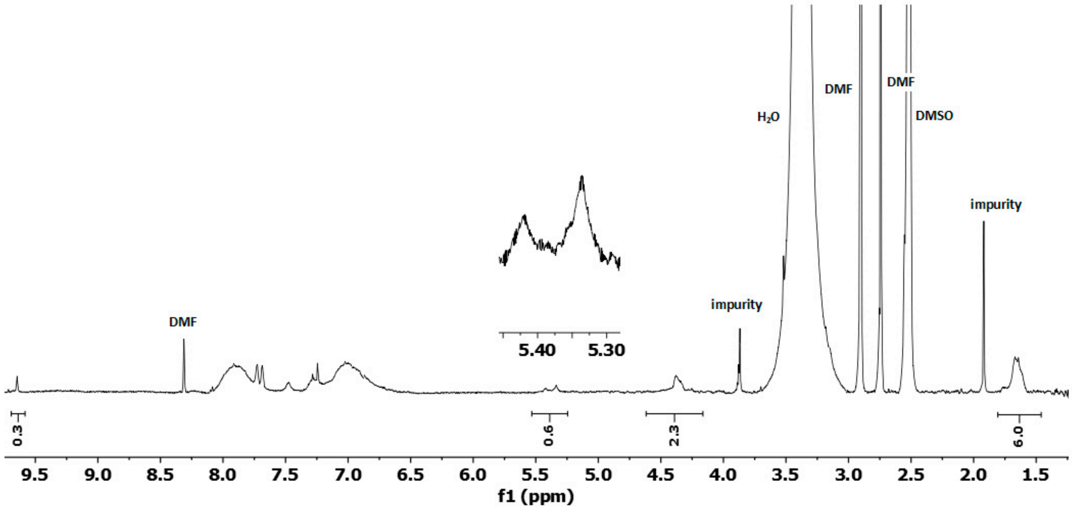

Figure S1. ^{1}H-NMR of POP-precursor in DMSO-d_6.

Typical signals of PSU are observed in the spectrum, particularly the methyl groups linked to quaternary carbon (1.6 ppm), the chloromethyl moiety at 4.4 ppm, and the aromatic region between 6.7-8.1 ppm. Furthermore, it is possible to recognize in the spectrum the peaks due to the ether linkage formed after the reaction with terephthalic ester (between 5.30-5.42 ppm, magnified in Fig. S1). The existence of two peaks can be related to the presence of two distinct isomers, derived by the reaction on the two different chloromethyl groups. The signals at 9.6 ppm are ascribed to the

phenolic proton of the terephthalic acid. Quoting 6 H for the methyl groups of PSU, the ether linkage amounts to 0.6 H, that is a functionalization degree of 0.3. The integration of the corresponding peak at 9.6 ppm is in accordance with the estimated degree of functionalization.

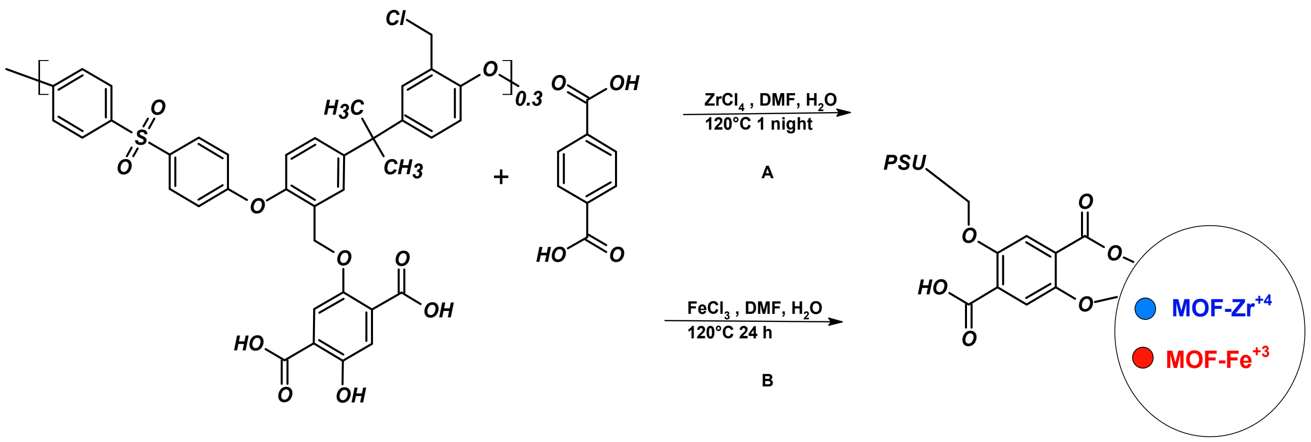

Scheme S2. Reaction pathways and schematic representation of (A) Zr-POP and (B) Fe-POP.

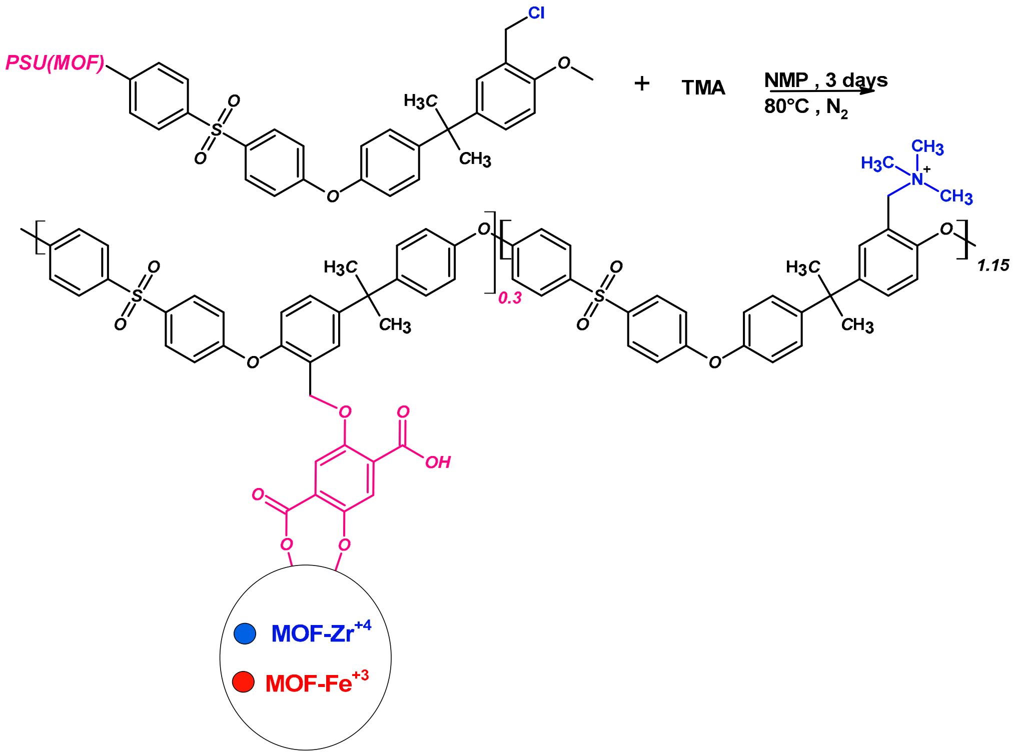

Scheme S3. Reaction pathways of Zr-POP-QA and Fe-POP-QA.

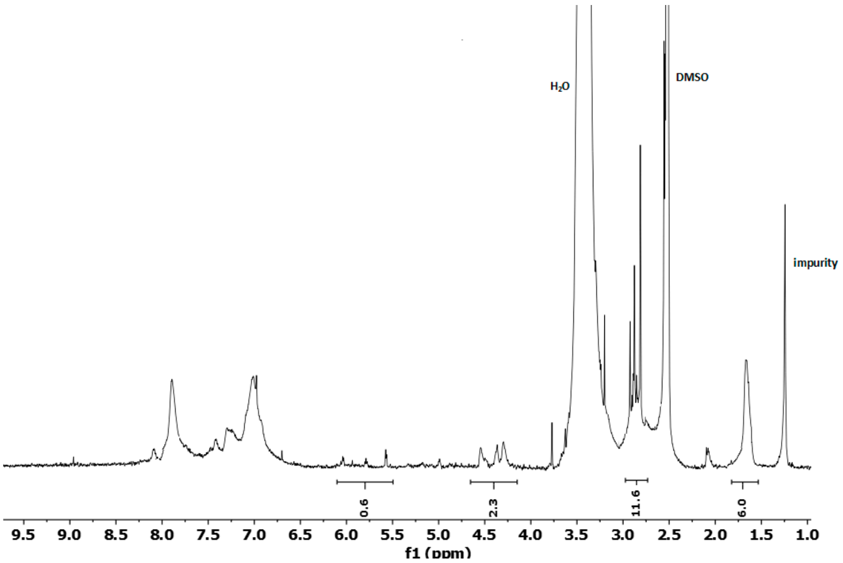

Figure S2. ^{1}H-NMR of Zr-POP-QA in DMSO-d_6.

After metal assembly, the signal ascribed to the ether is split into multiple peaks, between 5.5-6 ppm, indicating different conformations. Similarly, the -C<u>H</u>$_2$-N$^+$(CH$_3$)$_3$ signal is divided into 3 peaks centered at 4.4 ppm. The CH_3 linked to the ammonium is observed at approximately 2.9 ppm, also divided into 3 peaks. The integration is consistent with this interpretation and with the spectrum of Fig. S1. The area of the ether bonds related to the methyl groups of PSU remains constant, as well as the area of benzyl ammonium groups derived from the chloromethyl groups. The area of -CH$_2$-N(<u>CH</u>$_3$)$_3$ moieties is a little overestimated, probably affected by the presence of the signals of DMSO and water.

FTIR

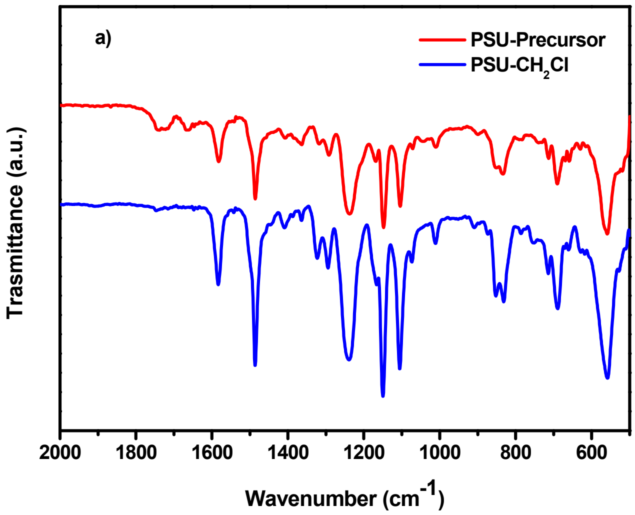

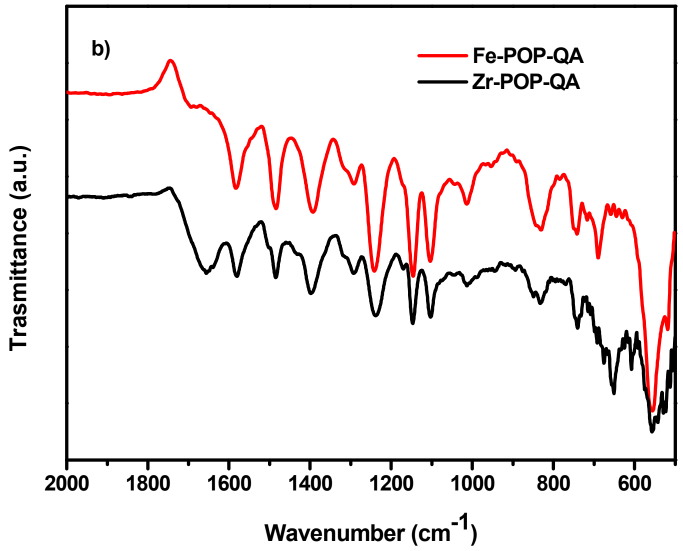

Figure S3. FTIR spectra of a) PSU-Precursor and PSU-CH_2Cl; b) Zr-POP-QA and Fe-POP-QA.

The peak at 1730 cm^{-1} (Figure S3a) is due to C=O stretch of terephthalic acid. The signal at 1670 cm^{-1} is only present in the PSU-precursor and can be assigned to the carboxylate anion. Other signals are due to the typical absorptions of PSU and terephthalic acid: at 1584 and 1488 cm^{-1} the skeletal vibration of aromatic hydrocarbons, at 1412, 1390, and 1365 cm^{-1} the benzene mode and CH_3 deformation of the polysulfone backbone, at 1325 and 1295 cm^{-1} the SO_2 asym stretching, at 1150 cm^{-1} the SO_2 sym stretching, at 1246 cm^{-1} the asym vibration of the ether linkage.

XPS

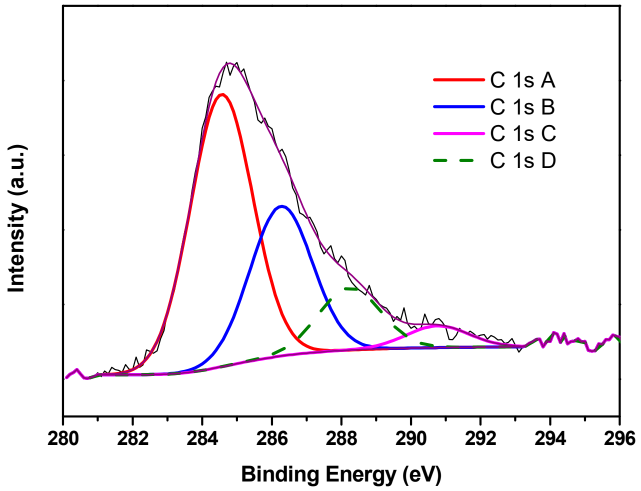

Figure S4. XPS spectra for C1s region of Zr-POP-QA.

C1s region is deconvoluted in three components: A at 284.6 eV (C=C, C-C), B at 286.3 eV (C-O), C 288.1 eV (-C=O, C-N), D 290.4 eV (-COO).

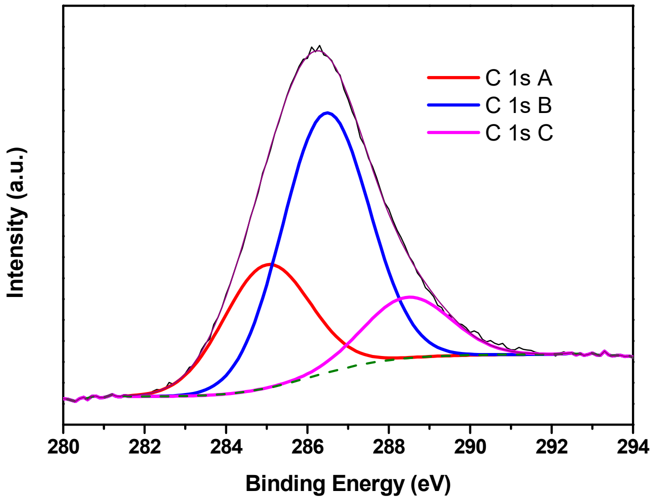

Figure S5. XPS spectra for C1s region of Fe-MOF-PSU-TMA.

C1s region is deconvoluted in three components: A, B, and C at 285.0 (C=C, C-C), 286.4 (C-O), and 288.4 (C=O, C-N) eV respectively.

Optical Microscopy

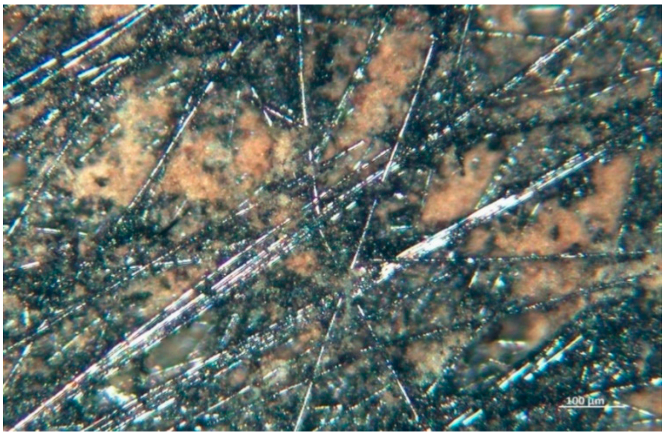

Figure S6. Optical Micrograph of Fe-POP-QA electrode on carbon paper.

BET analysis

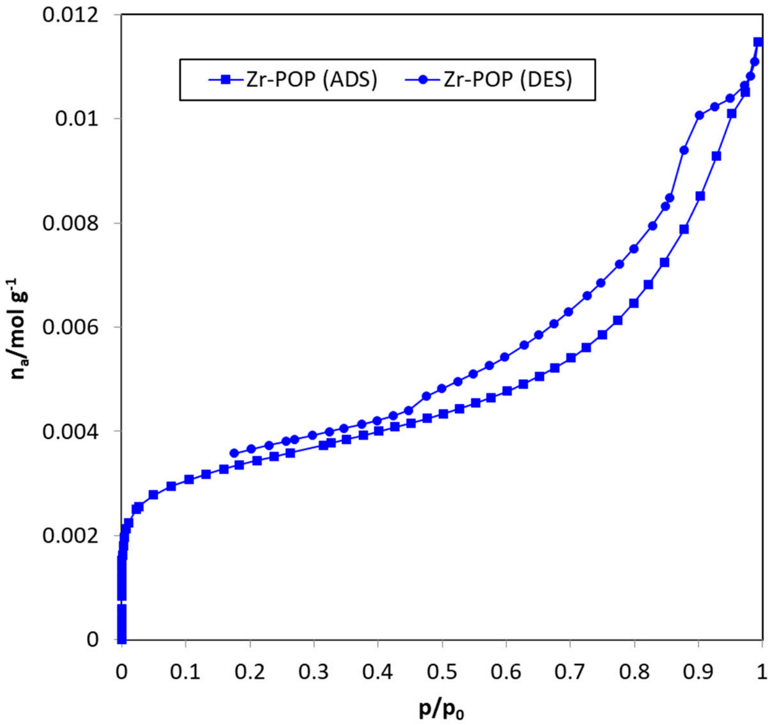

Figure S7. BET adsorption/desorption isotherm of Zr-POP.

The BET surface area of Zr-POP is 269 m^2/g; the average pore diameter is 5.8 nm.

Accelerated degradation tests

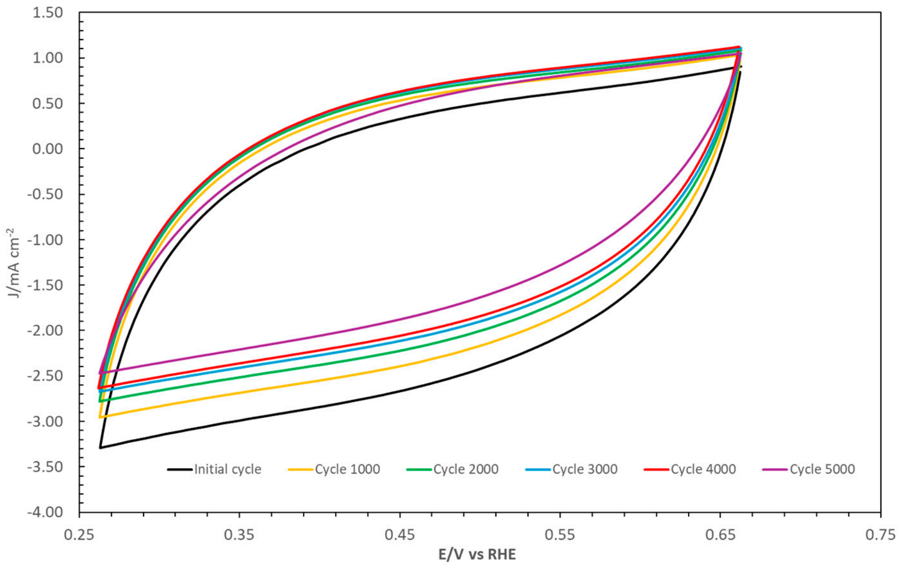

Figure S8. Accelerated degradation tests for Fe-POP-QA: cyclovoltammetric curves for the ORR in oxygen-saturated 0.1 M KOH solution at 1500 rpm RDE speed as function of the cycle number.

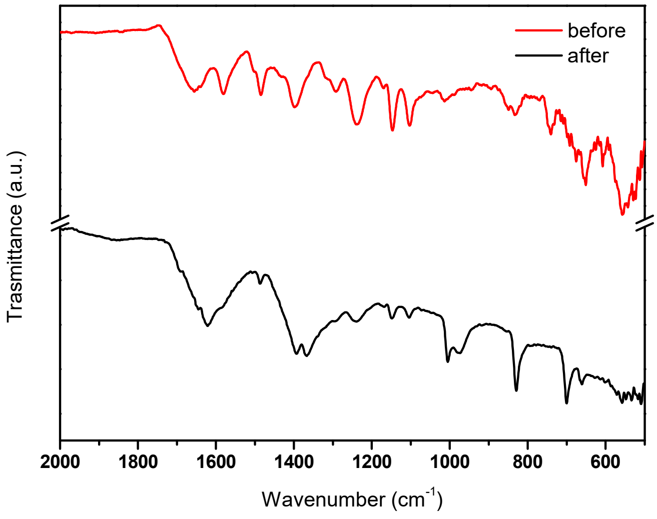

Figure S9. FTIR spectrum of Zr-POP-QA before and after the accelerated degradation test.

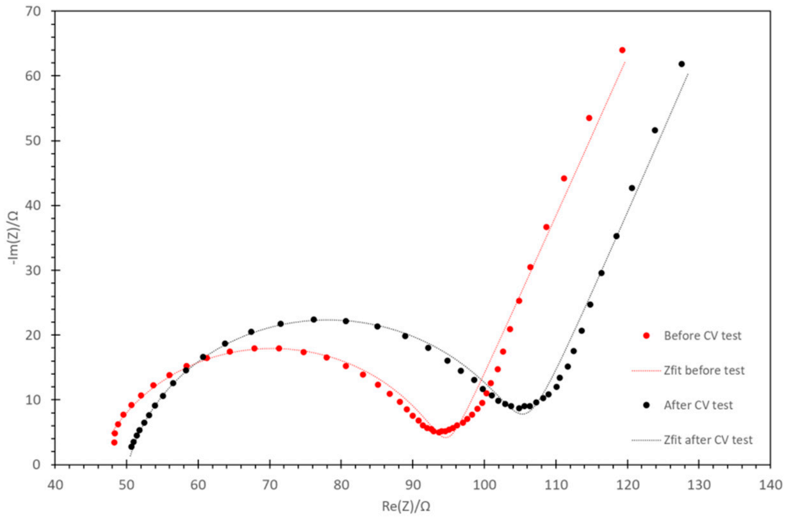

Figure S10. Impedance spectra of Zr-POP-QA electrodes before and after the accelerated degradation test.

The non-linear least-square fit parameters deduced from the impedance spectra are reported in Table S1. There is only a small increase of electrode/electrolyte resistance R1 and charge transfer resistance R2. The parameter Q3 is remarkably constant.

Table S1. Non-linear least-square fit parameters of Zr-POP-QA before and after the accelerated degradation test.

	R1/Ω	Q2/μFs$^{(n-1)}$	n2	R2/Ω	Q3/μFs$^{(n-1)}$	n3
before	45.7	1575	0.80	48.7	1101	0.75
after	50.1	3237	0.86	54.7	1116	0.76
